# Supplementary material for: Molecular mechanism for recognition of the cargo adapter Rab6GTP by the dynein adapter BicD2
Source: Life Sci Alliance. 2024 May 7;7(7):e202302430. doi: 10.26508/lsa.202302430 (PMC11077774; doi:10.26508/lsa.202302430)
Supplement: Supplementary file 2 [file LSA-2023-02430_TableS1.docx]

**Supplementary Information**

**Supplementary Tables**

**Table S1. List of Rab6^GTP^ interface**

**Residues with BicD2-CTD and ELKS**

**(yellow: shared contact residues).**

| ***Hs* BicD2**  **(Alphafold 2)** | **ELKS**  **PDB ID 8IJ9** |
| --- | --- |
|  | 8, 9, 11, 13, 14  21 - 22 |
| 35 |  |
| 43 - 50 | 42, 44, 46-50 |
|  | 51 |
| 52 | 52 |
| 54 | 54 |
|  | 56, 61 |
| 63 | 63 |
| 65 | 65 |
| 67 | 67 |
| 72, 74, 75 | 72 - 75 |
| 77 |  |
| 78 | 78 |
|  | 81 |
| 82 | 82  84, 85 |
|  | 96 – 99, 102 |
|  | 133, 135 |
