# Supplementary material for: Molecular mechanism for recognition of the cargo adapter Rab6GTP by the dynein adapter BicD2
Source: Life Sci Alliance. 2024 May 7;7(7):e202302430. doi: 10.26508/lsa.202302430 (PMC11077774; doi:10.26508/lsa.202302430)
Supplement: Supplementary file 4 [file LSA-2023-02430_TableS2.docx]

**Table S2. Summary of interactions between Rab6, GTP and Mg^2+^** (highlighted in Fig 2).

| **Rab6^GTP^/Q72L, PDB ID 2GIL** (Bergbrede et al, 2005) | | | |
| --- | --- | --- | --- |
| **Chain A** | **GTP, Mg^2+^** | | **Dist (Å)** |
| **Hydrogen bonds with GTP** | | | |
| ASP 129 OD1 | | GTP1200 N1 | 2.7 |
| ASP 129 OD2 | | GTP1200 N2 | 2.8 |
| TYR 42 OH | | GTP1200 O1G | 2.6 |
| THR 27 OG1 | | GTP1200 O2G | 3.9 |
| THR 45 N | | GTP1200 O2G | 3.0 |
| LYS 26 NZ | | GTP1200 O3G | 2.8 |
| GLY 71 N | | GTP1200 O3G | 2.9 |
| SER 23 N  LYS 26 NZ  LYS 26 N  VAL 24 N | | GTP1200 O3B  GTP1200 O1B  GTP1200 O1B  GTP1200 O1B | 3.0  2.6  2.9  3.4 |
| GLY 25 N | | GTP1200 O1B | 3.1 |
| THR 27 OG1  THR 27 N  VAL 24 N  THR 27 N  SER 28 N | | GTP1200 O2B  GTP1200 O2B  GTP1200 O3A  GTP1200 O1A  GTP1200 O1A | 2.9  3.1  3.7  3.6  2.9 |
| SER 28 OG | | GTP1200 O1A | 2.6 |
| LYS 127 NZ | | GTP1200 O4’ | 3.1 |
| ASN 126 ND2 | | GTP1200 O6 | 3.3 |
| ALA 157 N | | GTP1200 O6 | 2.9 |
| LYS 158 N | | GTP1200 O6 | 3.3 |
|  | | |  |
| **Interface residues of Rab6 with GTP**  22-28  38-45  69, 70, 71  126,127  129, 130  157, 158  **Interface residues of Rab6 with Mg^2+^**  26-27  42-45  69 | | | |
|  | | | |

Standard nomenclature from the structural coordinates is used for chain ID, residue names and atom names. Dist: Distance. Hydrogen bonds and contact residues were identified with the PISA server (Krissinel & Henrick, 2007).
