# Supplementary material for: Molecular mechanism for recognition of the cargo adapter Rab6GTP by the dynein adapter BicD2
Source: Life Sci Alliance. 2024 May 7;7(7):e202302430. doi: 10.26508/lsa.202302430 (PMC11077774; doi:10.26508/lsa.202302430)
Supplement: Supplementary file 5 [file LSA-2023-02430_TableS3.docx]

**Table S3 Secondary structure estimation from the CD spectra with BeStSel** (Micsonai *et al*, 2022).

The X-ray structure of human Rab6^GTP^/Q72L (residues 13-174, PDB ID 2GIL) consists of: 35.1% α-helix; 18.8% β-sheet. These percentages were calculated for the full-length protein with 208 residues (assuming disordered C- and N-termini), since the full-length protein was analyzed by CD spectroscopy.

| **Rab6^GTP^ mutant** | **α-helix (%)** | **Anti-parallel β-sheet (%)** | **Parallel β-sheet (%)** | **Turn (%)** | **Other (%)** |
| --- | --- | --- | --- | --- | --- |
| **WT** | 21.8 | 14.7 | 4.3 | 15.7 | 43.4 |
| **F75A** | 20.5 | 18.2 | 5.1 | 15.3 | 40.9 |
| **Y82A** | 20.3 | 10.6 | 3.4 | 13.7 | 52.1 |
| **I79A** | 20.4 | 13.5 | 5.0 | 13.1 | 48.0 |
| **P80A** | 21.2 | 10.1 | 4.4 | 13.6 | 50.7 |
| **I83A** | 20.2 | 9.8 | 5.8 | 13.8 | 50.4 |
| **Y42A** | 22.0 | 12.9 | 6.8 | 13.9 | 44.4 |
| **I46A** | 23.0 | 11.6 | 4.2 | 15.0 | 46.2 |
| **D49A** | 20.9 | 11.9 | 5.7 | 14.1 | 47.5 |
| **F50A** | 22.4 | 12.5 | 2.1 | 16.1 | 46.9 |
| **F38A** | 22.2 | 11.0 | 6.3 | 14.5 | 46.0 |
| **T45A** | 22.0 | 10.1 | 8.4 | 14.0 | 45.5 |
| **K53A** | 21.7 | 11.3 | 4.6 | 15.0 | 47.4 |
| **R63A** | 23.8 | 10.3 | 4.7 | 13.9 | 47.2 |
| **Q65A** | 22.5 | 10.3 | 5.3 | 13.9 | 47.9 |
| **W67A** | 22.4 | 14.6 | 4.0 | 16.0 | 43.0 |
| **T69A** | 21.6 | 13.8 | 6.4 | 15.4 | 42.8 |
| **Y35A** | 21.4 | 12.9 | 5.6 | 13.9 | 46.3 |
| **T54A** | 23.8 | 11.2 | 4.5 | 14.7 | 45.8 |
| **L78A** | 21.5 | 16.0 | 3.8 | 15.7 | 42.9 |
| **Rab6GDP** | 23.5 | 10.8 | 3.2 | 13.9 | 48.6 |
| **Rab6 nucleotide free** | 25.1 | 11.9 | 4.3 | 15.6 | 43.1 |
