# Supplementary material for: Molecular mechanism for recognition of the cargo adapter Rab6GTP by the dynein adapter BicD2
Source: Life Sci Alliance. 2024 May 7;7(7):e202302430. doi: 10.26508/lsa.202302430 (PMC11077774; doi:10.26508/lsa.202302430)
Supplement: Supplementary file 6 [file LSA-2023-02430_TableS4.docx]

| **Table S4 Summary of contact residues from binding assays, CD spectroscopy and Alphafold2** | | | |  |
| --- | --- | --- | --- | --- |
| ***Hs* Rab6 residue #** | **Reduced binding?*** | **Folded in CD?**** | **Contact residue***** |  |
|  | **√** | **√** | **√** |  |
| Q22 | x |  | x |  |
| S23 | x |  | x |  |
| 24-34 |  |  | x |  |
| Y35 | √ | √ | √ |  |
| D36 |  |  | x |  |
| S37 |  |  | x |  |
| F38 | √ | √ | x |  |
| D39 |  |  | x |  |
| N40 |  |  | x |  |
| T41 |  |  | x |  |
| Y42 | √ | √ | x |  |
| Q43 | x |  | √ |  |
| A44 |  |  | √ |  |
| T45 | √ | √ | √ |  |
| I46 | √ | √ | √ |  |
| G47 |  |  | √ |  |
| I48 | x |  | √ |  |
| D49 | √ | √ | √ |  |
| F50 | √ | √ | √ |  |
| L51 | x |  | x |  |
| S52 | x |  | √ |  |
| K53 | √ | √ | x |  |
| T54 | √ | √ | √ |  |
| 55-62 |  |  | x |  |
| R63 | √ | √ | √ |  |
| L64 |  |  | x |  |
| Q65 | √ | √ | √ |  |
| L66 |  |  | x |  |
| W67 | √ | √ | √ |  |
| D68 |  |  | x |  |
| T69 | √ | √ | x |  |
| A70 |  |  | x |  |
| G71 |  |  | x |  |
| Q72 |  |  | √ |  |
| E73 |  |  | x |  |
| R74 | x |  | √ |  |
| F75 | √ | √ | √ |  |
| R76 |  |  | x |  |
| S77 | x |  | √ |  |
| L78 | √ | √ | √ |  |
| I79 | √ | √ | x |  |
| P80 | √ | √ | x |  |
| S81 | x |  | x |  |
| Y82 | √ | √ | √ |  |
| I83 | √ | √ | x |  |
| R84 | x |  | x |  |
| D85 | x |  | x |  |
| 86-87 |  |  | x |  |

*Results from pulldown assays, Fig 2 and Fig S7. **Results from CD spectroscopy,

see Fig 2 and Fig S8. ***Results from the Alphafold 2 model, see Fig 1 and Fig S3D.
